# Supplementary material for: Cost-Effective Detection of Perfluoroalkyl Carboxylic Acids with Gas Chromatography: Optimization of Derivatization Approaches and Method Validation
Source: Int J Environ Res Public Health. 2019 Dec 21;17(1):100. doi: 10.3390/ijerph17010100 (PMC6982026; doi:10.3390/ijerph17010100)
Supplement: Supplementary file 1 [file ijerph-17-00100-s001.pdf]

Supplementary Materials for:

Cost-effective Detection of Perfluoroalkyl Carboxylic Acids with Gas Chromatography: Optimization of Derivatization Approaches and Method Validation

Zhen Li[1, 2], *Hongwei Sun*[1, 3, *]

[1] Key Laboratory of Pesticide & Chemical Biology of Ministry of Education, Institute of Environmental & Applied Chemistry, College of Chemistry, Central China Normal University, Wuhan 430079, PR China.

[2] Hubei High-tech Innovation and Business Incubation Center, Wuhan 430000, PR China.

[3] Division of Environmental Science and Engineering, Pohang University of Science and Technology (POSTECH), Pohang 37673, South Korea.

* Corresponding author. E-mail address: rzhdw@postech.ac.kr (H. Sun)

Table S1 The composition of the surface water sample collected from the local lake

**parameter**	**value**
Conductivity (mS/cm)	0.39
Salinity (ppt)	0.18
Total suspended solid (g/L)	0.26
Dissolved oxygen (mg/L)	7.93
Redox potential (mV)	71.05
pH	7.55
Temperature (°C)	21
Total phosphorus (mg/L)	0.81
COD (mg/L)	18.64

Table S2 The L_{18} (3^7) orthogonal experiment design which shows the combination of different levels of factors

**Experimental No.**	**A**	**B**	**C**	**D**	**E**	**F**	**G**
**1**	1	1	1	1	1	1	1
**2**	1	2	2	2	2	2	2
**3**	1	3	3	3	3	3	3
**4**	2	1	1	2	2	3	3
**5**	2	2	2	3	3	1	1
**6**	2	3	3	1	1	2	2
**7**	3	1	2	1	3	2	3
**8**	3	2	3	2	1	3	1
**9**	3	3	1	3	2	1	2
**10**	1	1	3	3	2	2	1
**11**	1	2	1	1	3	3	2
**12**	1	3	2	2	1	1	3
**13**	2	1	2	3	1	3	2
**14**	2	2	3	1	2	1	3
**15**	2	3	1	2	3	2	1
**16**	3	1	3	2	3	1	2
**17**	3	2	1	3	1	2	3
**18**	3	3	2	1	2	3	1

Table S3 The instrumental LODs of PFCAs anilides detected by GC-μECD and the parameter values used for the calculation of LODs.

	residual standard deviation	standard deviation of intercept	slope	LOD based on residual SD (μg L^{-1})	LOD based on intercept SD (μg L^{-1})
PFBA	162.93	87.60	253.48	2.12	1.14
PFPeA	178.56	96.01	193.71	3.04	1.64
PFHxA	265.83	142.93	228.10	3.85	2.07
PFHpA	106.27	57.14	135.07	2.60	1.40
PFOA	124.94	67.18	94.17	4.38	2.35
PFNA	459.58	247.11	129.37	11.72	6.30
PFDeA	75.97	40.85	82.40	3.04	1.64
PFUnA	40.83	21.95	62.15	2.17	1.17
PFDoA	237.70	127.81	66.70	11.76	6.32

Table S4 Cost evaluation of the derivatization-GC method for the analysis of PFCAs in comparison with the HPLC-MS/MS method.

**Reagent and instrument**	**Cost per sample (USD)**	
	**GC method**	**HPLC-MS/MS**
1mL 1 N HCl	4.06E-04	
0.2g NaCl	7.54E-04	
5 mL ethyl acetate	4.17E-02	
0.4 mL 0.1 M 2,4-difluoroaniline	8.23E-03	
0.2 mL 0.1 M N,N-dicyclohexylcarbodiimide	6.70E-04	
1g NaCl	3.77E-03	
3 mL ethyl acetate	2.50E-02	
5 mL 1 N HCl	2.03E-03	
5mL $NaHCO_3$ (100g/L)	5.31E-03	
5mL NaCl (500g/L)	9.42E-03	
Na_2SO_4	4.49E-05	
GC	5.76-14.40	
HPLC-MS/MS		43.21-72.01
**Total**	5.86-14.50	43.21-72.01

Table S5 The comparison of this study and the method reported by Scott and coworkers (Environ. Sci. Technol. 2006, 40, 20, 6405-64100).

	Scott and coworkers	this study
Water sample pretreatment procedure	1. 1 L water sample reduced to 50 mL by rotary evaporator at 40 °C<br>2. 50 L sample was passed through precleaned XAD-7 resin, then the resin was extracted with methanol. The methanol was evaporated to dryness, then 50 mL water and 35 mL ethyl acetate was added for derivatization	WAX cartridges conditioned by 6 mL of 0.1% NH_4OH methanol solution, 6 mL methanol and 6 mL distilled water → 1 L water were loaded→washed with 6 mL 2.5 M ammonium acetate buffer (pH=4) → Analytes eluted by 10 mL 0.1%, NH_4OH methanol → remove of methanol and solvent exchange to water (5 mL) by nitrogen stream→derivatization
Derivatizing procedure	50 mL water sample → pH adjusted to 1.0 →add 1 g NaCl, 35 mL ethyl acetate → add 0.4 mL 0.1 M 2,4-difluoroaniline, 0.4 mL 0.1 M N, N-dicyclohexylcarbodiimide → stir for 1 h → add 5 g NaCl → collect ethyl acetate phase → aqueous phase re-extracted with 15 mL ethyl acetate→ combined ethyl acetate extracts sequentially washed with 5 mL of 1 N HCl, saturated $NaHCO_3$ and NaCl solutions → rotary evaporated to dryness → add 10 mL 1.5% propanol in toluene → purified with 2.1g activated silica gel →evaporated under nitrogen gas flow to 2 mL	5 mL water sample → pH adjusted to 1.0 →add 0.2 g NaCl, 5 mL ethyl acetate → add 0.2 mL 0.1 M 2,4-difluoroaniline, 0.4 mL 0.1 M N, N-dicyclohexylcarbodiimide → shaken for 0.5 h at 200 rpm under ambient temperature → add 1 g NaCl → collect ethyl acetate phase → aqueous phase re-extracted with 3 mL ethyl acetate→ combined ethyl acetate extracts sequentially washed with 1 mL of 1 N HCl, saturated $NaHCO_3$ and NaCl solutions → dehydrated with anhydrous Na_2SO_4 →evaporated under nitrogen gas flow to 0.5 mL
Instrumental analysis	GC-MS	GC-μECD
Detection limit (based on 1L sample)	0.5 ng/L based on 1 L sample<br>0.01 ng/L based on 50 L sample (XAD-7 method)	0.1-0.6 ng/L based on 1 L sample and final volume of 0.1 mL for injection
Recovery	C7-C9 PFCAs: 60%-160%<br>C6 PFCAs: 25%-35%<br>Other PFCAs: not available	C4-C12 PFCAs: 62%-118% for spiked blanks and 57%-117% for spiked matrices

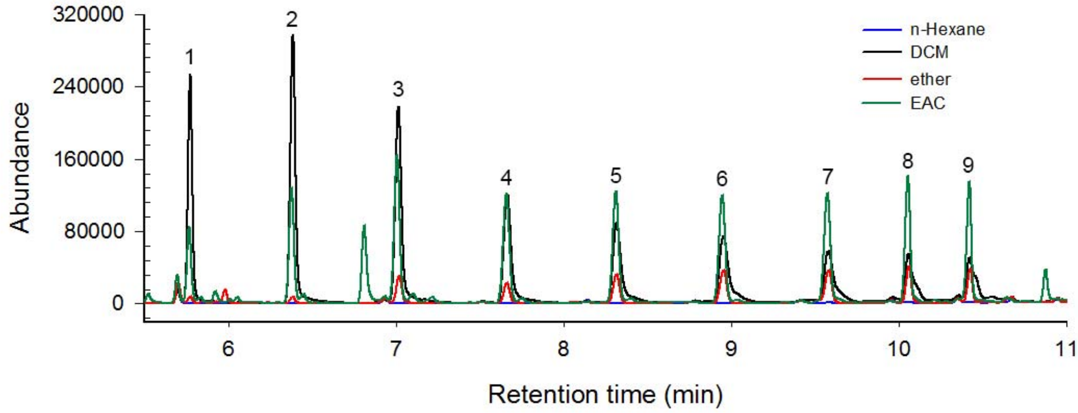

Fig. S1 Chromatograms of PFCAs anilides generated by using different co-solvent for derivatization, namely n-hexane, dichloromethane (DCM), ethyl ether and ethyl acetate (EAC). Peak identification: (1) PFBA, (2) PFPeA, (3) PFHxA, (4) PFHpA, (5) PFOA, (6) PFNA, (7) PFDA, (8) PFUnA and (9) PFDoA.

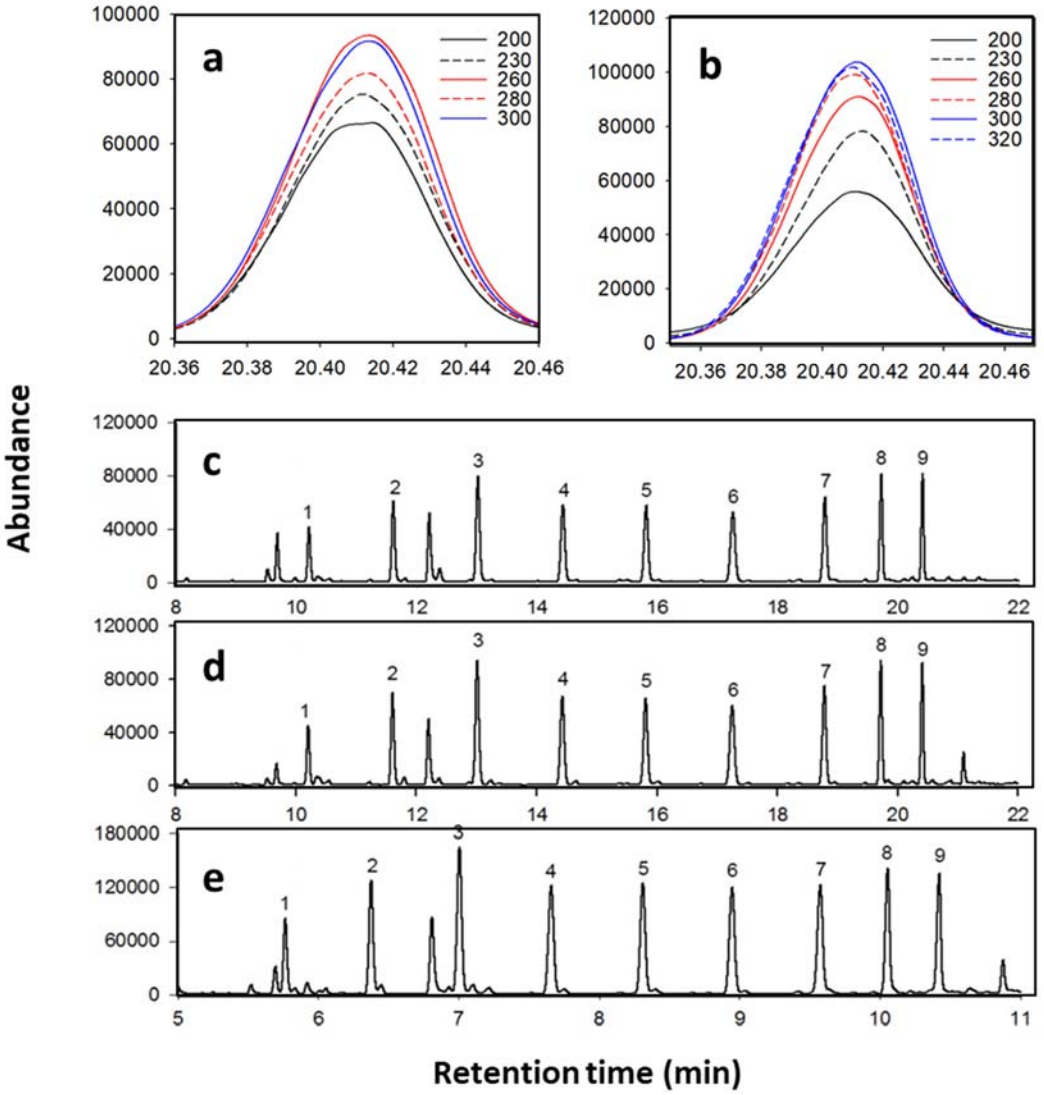

Fig. S2 The GC chromatograms of PFNA anilides with (a) different inlet temperatures, and (b) detector temperatures; Chromatograms of PFCAs anilides under different oven temperature programs: (c) TP1, (d) TP2, and (e) TP3. The details of TP1, TP2 and TP3 are described in Section 2.5, and the peaks identification in (c-e) is: (1) PFBA, (2) PFPeA, (3) PFHxA, (4) PFHpA, (5) PFOA, (6) PFNA, (7) PFDA, (8) PFUnA and (9) PFDoA.

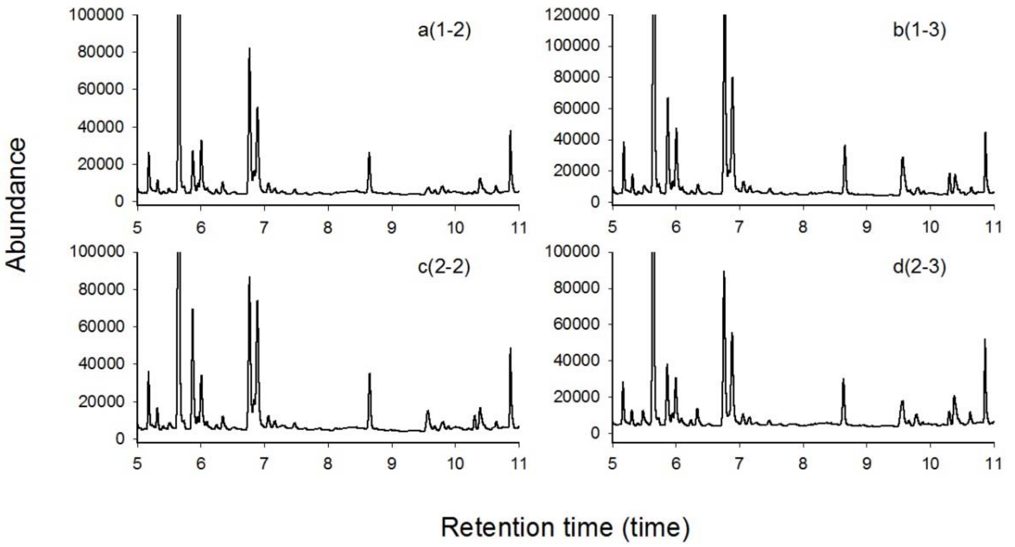

Fig. S3 GC chromatograms of PFCAs in the fractions sequentially eluted from the SPE cartridges using different concentrations of NH_4OH. a(1-2): SPE cartridge conditioned with 0.1% NH_4OH methanol solution and eluted with 1% NH_4OH; b(1-3): conditioned with 0.1% NH_4OH methanol solution and eluted with 10% NH_4OH; c(2-2): conditioned with 1% NH_4OH methanol solution and eluted with 1% NH_4OH; d(2-3): conditioned with 1% NH_4OH methanol solution and eluted with 10% NH_4OH

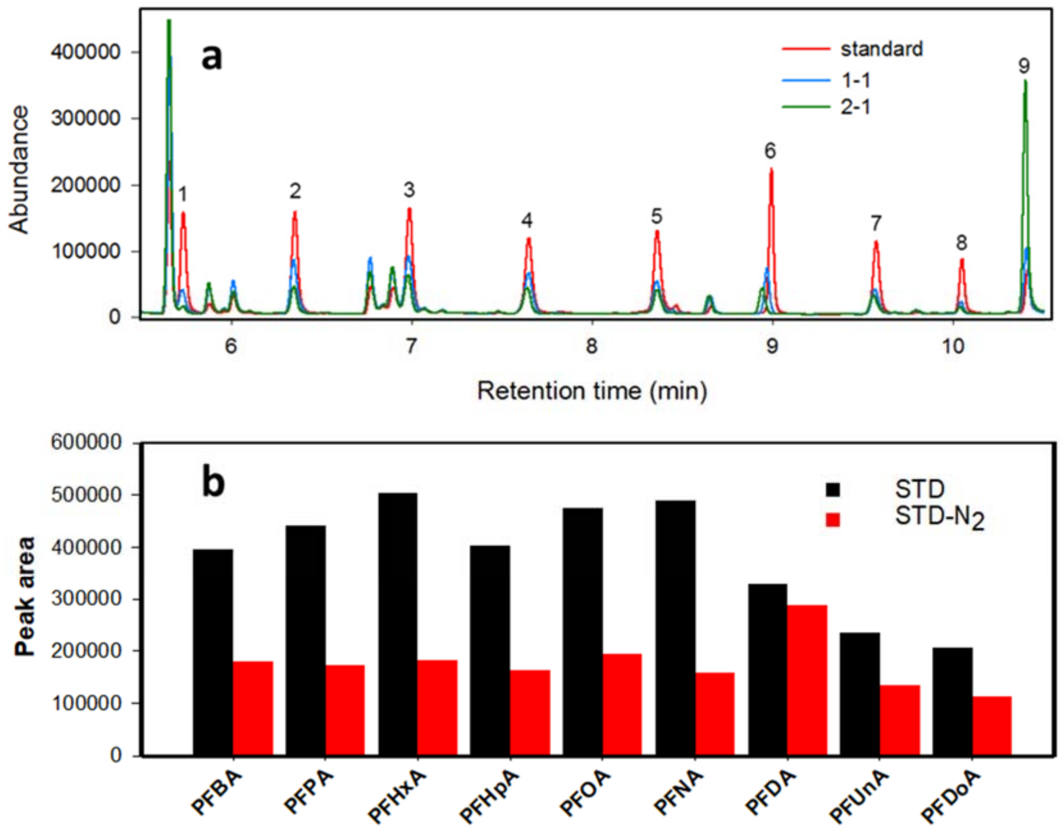

Fig. S4 (a) Comparative chromatograms of PFCA anilides derived from spiked standard sample and SPE extracted samples. 1-1: SPE cartridges conditioned by 0.1 % NH_4OH methanol solution and eluted by the same solution; 2-1: SPE cartridges conditioned by 1 % NH_4OH methanol solution and eluted by 0.1 % NH_4OH methanol solution; the peak identification is the same with previous chromatograms. (b) Impact of concentrating step by nitrogen gas flow on the peak areas of target PFCA anilides during the pretreatment procedure of spiked water samples; STD or STD-N_2: PFCAs standards derived *w/o* or *w/* nitrogen evaporation step.
